# A Systematic Review of Behavioural Interventions Promoting Healthy Eating among Older People

**DOI:** 10.3390/nu10020128

**Published:** 2018-01-26

**Authors:** Xiao Zhou, Federico J. A. Perez-Cueto, Quenia Dos Santos, Erminio Monteleone, Agnès Giboreau, Katherine M. Appleton, Thomas Bjørner, Wender L. P. Bredie, Heather Hartwell

**Affiliations:** 1 Department of Food Science, University of Copenhagen, 1958 Frederiksberg C, Denmark; xiao.zhou@food.ku.dk (X.Z.); quenia@food.ku.dk (Q.D.S.); wb@food.ku.dk (W.L.P.B.); 2 Department of Management of Agricultural, Food and Forestry Systems, University of Florence, 50144 Florence, Italy; erminio.monteleone@unifi.it; 3 Centre for Food and Hospitality Research, Institute Paul Bocuse, 69130 Ecully, France; agnes.giboreau@institutpaulbocuse.com; 4 Research Centre for Behaviour Change, Department of Psychology, Faculty of Science and Technology, Bournemouth University, Poole BH12 5BB, UK; k.appleton@bournemouth.ac.uk; 5 Department of Architecture, Design and Media Technology, Aalborg University, 9000 Aalborg, Denmark; tbj@create.aau.dk; 6 Faculty of Management, Bournemouth University, Poole BH12 5BB, UK; hhartwell@bournemouth.ac.uk

**Keywords:** behavioural intervention, healthy eating, older people, systematic review

## Abstract

Because eating habits are inseparably linked with people’s physical health, effective behaviour interventions are highly demanded to promote healthy eating among older people. The aim of this systematic review was to identify effective diet interventions for older people and provide useful evidence and direction for further research. Three electronic bibliographic databases—PubMed, Scopus and Web of Science Core Collection were used to conduct a systematic literature search based on fixed inclusion and exclusion criteria. English language peer-reviewed journal articles published between 2011 and 2016 were selected for data extraction and quality assessment. Finally, a total of 16 studies were identified. The studies’ duration ranged from three weeks to seven years. The majority of studies were carried out in European countries. Seven studies had a moderate quality while the remaining studies were at a less than moderate level. Three dietary educational interventions and all meal service related interventions reported improvements in older people’s dietary variety, nutrition status, or other health-related eating behaviours. Multicomponent dietary interventions mainly contributed to the reduction of risk of chronic disease. The results supported that older people could achieve a better dietary quality if they make diet-related changes by receiving either dietary education or healthier meal service. Further high-quality studies are required to promote healthy eating among older people by taking regional diet patterns, advanced information technology, and nudging strategies into account.

## 1. Introduction

According to a report from the United Nations, the proportion of people worldwide aged 60 years and over is predicted to increase by 56% between 2015 and 2030 [1]. With respect to the health problems caused by aging processes, people are aware of the importance of good health and high quality of life in one’s later life [2]. A series of health problems may arise when people become older, such as chronic diseases [3], malnutrition [4], and falls [5]. Take malnutrition for instance, as a frequent complication, it has a strong relationship with the older person’s ill-health and disability [6]. Weight imbalance is also related to the older person’s physical function and influences the quality of life [7,8]. Underweight and obesity as worldwide challenges are positively associated with mortality during the aging processes [9,10]. Therefore, it is highly recommended that older people should be given effective interventions to enhance their health status and improve their living quality.

Food as a daily necessity plays a vital role in people’s life. A variety of nutrients and functional compounds can be obtained from vegetables and fruits, such as vitamins, fibres, minerals, polyphenols, and flavonoids [11]. Intakes of healthy food, especially vegetables and fruits have been proved beneficial to physical health [12,13,14]. Scientific research has shown that extracts from vegetable and fruit can help to prevent or alleviate older people’s illness and suffering [15,16,17,18]. Therefore, it can be a feasible approach to improve the quality of later life by encouraging older people to intake more vegetables and fruits and change their eating habit towards a healthier level.

Dietary intervention as an effective method has contributed substantially to delay or prevent diseases among older people [19,20,21]. However, interventions with the aim of increasing vegetable intake for this age group have been notably omitted from study groups [22]. Fragile condition, social, and physiological changes during the aging processes may influence older consumers’ eating behaviour [23]. For instance, older people with chewing and swallowing difficulties had a lower micro- and macronutrient intake from food, and hence demonstrated a declined nutrition status [24]. In addition, weakened taste ability and declined olfactory function had an impact on older people’s appetite, food choice, and intake [24]. Therefore, effective and advanced dietary interventions on promoting heathier eating for older people are highly in demand.

Substantial studies of promoting older people’s healthy eating can present evidence and provide recommendations to update current dietary interventions. Previous systematic reviews have shown that dietary interventions such as nutrition education and counselling, or enriching a standard diet with energy and protein powder, had somehow positive effects on older people’s actual eating behaviour or physical conditions. However, some included studies in these systematic reviews involved nutrition supplements and did not purely address older people’s daily diet [25,26]. Therefore, this systematic review was conducted to collect the latest strategies regarding the promotion of healthy eating among older people, and to give further possible directions for future improvement.

## 2. Materials and Methods

This systematic review was conducted following the guideline of PRISMA and has been registered on PROSPERO (registration number is CDR 42016042682).

### 2.1. Literature Search Strategy

Electronic bibliographic databases—PubMed, Scopus, and Web of Science Core Collection were used for conducting this literature search. The search strategy was based on a clear and careful selection of key words and terms. After repeated attempts and adjustments, the final search strategy was built and is shown as follows: intervention* AND (diet* OR nutrition OR healthy eating) AND (elder* OR senior* OR old*). To collect the latest studies, each study’s publication time was restricted to range from January 2011 to February 2016. The search filter was set on human beings and language was restricted to English. References from reviews and systematic reviews were also checked manually for further screening in case they were not identified during the whole search process.

### 2.2. Inclusion and Exclusion Criteria

The included studies had to be behavioural interventions with the aim of changing older people’s eating habits or improving physical health by serving healthy food. For instance, interventions such as providing dietary education or meal service on promoting older people’s healthy eating were included. Normally, the age of older people is defined as 60-year-old and over according to the United Nations’ report [27], however, considering the heterogeneous definition for older adults [28], studies targeting people of 50–60 years old were also included as they categorized the subjects as ‘elderly’ or ‘older’. Interventions that took place at residential homes, nursing homes, study centres, or hospitals were included in this systematic review. Subjects with a high risk factor of chronic disease were also included. Regarding the whole environment interactions, interventions involving meal provider or day care nurses were also included. In addition, all the included studies should have follow-up visiting. Considering the study type, experimental studies such as randomized controlled trials and quasi-experiments were included.

Studies were excluded if the target group were children, youths, and adults. Besides, studies focusing on small simple size (<20) [29] were excluded from this review because of low statistical power. Older people with cancer, dementia, tube-feeding, or at a terminal stage were not considered on account of their unstable and uncontrolled eating behaviour. Dietary intervention involving supplements such as vitamins or fortified foods were also eliminated. Furthermore, observational studies such as cross-sectional studies were excluded from this systematic review. Studies containing physical activity interventions were excluded unless it was analysed separately from diet intervention. Book chapters and descriptive articles were not considered in this systematic review.

### 2.3. Screening

For the primary step, duplicates were identified and eliminated by using the reference management tool—EndNote X7.0.1 (Clarivate Analytics, Philadelphia, PA, USA). Then, studies against inclusion criteria or within exclusion criteria were removed by reading titles and abstracts, which were completed by two reviewers. Further screening of studies was conducted by reading the full text of the paper according to the criteria.

### 2.4. Data Extraction

A predetermined grid was adopted to perform data extraction, including the following information: author, publication year, country, age, setting, sample size, study design, description of intervention, comparison, duration, measurement, and main outcome. This step was completed by two reviewers independently through full-text reading. The extracted data used for quality assessment were further analysed with a third reviewer and finalized by consensus.

### 2.5. Quality Assessment

Following Cochrane’s guideline, risk of bias was assessed by at least two reviewers independently, then the agreed assessment was further entered into the software Review Manager 5.3.5 (The Nordic Cochrane Centre, Copenhagen, Denmark). If agreement was not achieved, a third reviewer would contribute to the assessment. In total, there are seven domains for quality assessment: (1) Random sequence generation; (2) Allocation concealment; (3) Blinding of participants and personnel; (4) Blinding of outcome assessment; (5) Incomplete outcome data; (6) Selective reporting; (7) Other bias (other source of bias could put the study at a high risk of bias in certain circumstances, e.g., carry-over in cross-over trials, baseline imbalance). Each judgement has three options: low risk, high risk, and unclear risk. Consequently, two figures were generated by the software to present the risk of bias in the selected studies [30].

### 2.6. Data Analysis

Because of the high heterogeneity of the studies’ measures and the limited articles, a narrative synthesis was performed. Meta-analysis was unfeasible to run as the measurement units of each study were not comparable. Therefore, a narrative synthesis was conducted concentrating on the general characteristic of included studies, participants, intervention type, study quality, and the reported effects of the interventions on older people’s eating behaviour and health condition.

## 3. Results

### 3.1. Study Selection

**Figure 1:**
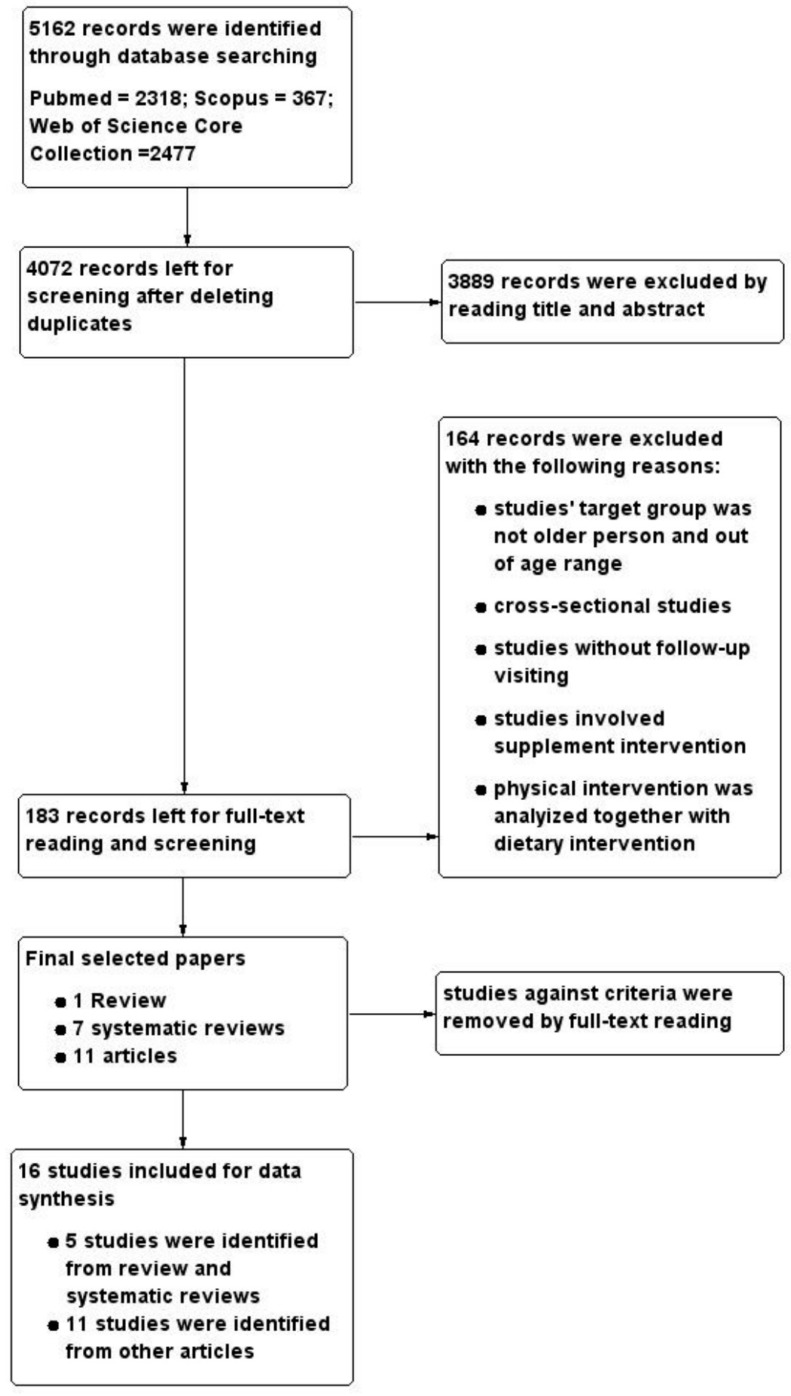
PRISMA diagram showing the screening process.

A total of 5162 results were obtained from three databases after applying the predefined search strategy (see Section Section 2.1). After removing duplicates, 4072 papers were left for screening. Initially, 183 papers were retained after checking each paper’s title and abstract. After full-text reading and screening, the remaining 19 articles (one literature review, seven systematic reviews, and eleven articles) were retrieved, read, and studies that met the inclusion criteria were retained for data extraction. An additional five relevant studies were identified by checking the references from included reviews and added to the final analysis. In total, 16 unique studies were selected for data extraction and data analysis. Figure 1 shows the progress of screening.

### 3.2. Study Characteristics

Table 1 shows the characteristics of the included studies.

Publication date was limited to the last five years. Among the included 16 studies, only one was published in 2014 and one in 2015 [41,44]. The majority of the studies were carried out in European countries [20,21,31,32,34,35,37,38,40,41,42,44], with Spain as the most frequent country. Only two studies were performed in the USA [39,43], while two others were in countries from Asia [33,36]. Main intervention settings were research centre [20,31,34,42,43,44], residential home [21,32,40], primary care centre [38,41], social group [35,37], community centre [33], and elderly centre [36]. Only one article [39] that reported the setting was arranged in two separate places, with the purpose of making comparisons between a congregate area and residential home. Two articles reported that the target area was a rural county [39,43]. Nine articles reported approval from an ethical committee [21,32,33,34,35,36,37,40,44], however, only three of them provided citations [32,40,44].

Regarding the study design, this systematic review included ten randomized controlled trials (RCTs) [20,21,31,33,34,38,41,42,43,44], four quasi-experimental interventions [35,36,39,40], one within-subjects design [32], and one quasi-RCT [37]. In addition, only one study was reported as a pilot trial [34].

The number of participants from each study varied considerably, ranging from 23 to 3923. Participants’ minimum age was 50 years and maximum age was 90 years. Considering gender differences, women were the majority participants and one study only targeted females [43]. Five studies investigated the effect of dietary intervention on participants with risk factors of chronic disease [20,31,38,41,42], one study focused on frail older people by giving nutrition counselling [34], and two articles analysed the effect of food-related interventions on older consumers’ vegetable and fruit intake [21,37].

Three types of interventions were identified based on the approaches: dietary educational intervention, meal service intervention, and multicomponent intervention. Seven studies were identified as dietary educational interventions because they conducted a health program, provided a mailed tailored newsletter, or nutrition advice for older participants [33,34,35,36,39,43,44]. Meal service interventions in four studies consisted of either healthy food serving or repeated exposure to food [21,31,32,37]. Five studies had a multicomponent design as they provided both a healthy meal and additional dietitian’s advice [20,31,38,41,42]. The duration of the included studies showed notable differences, ranging from three weeks to seven years. More than half of them lasted at least one year [20,31,32,34,35,38,39,40,41,42,43], while only three studies took around one month [36,37,44].

### 3.3. Study Quality Assessment

**Figure 2:**
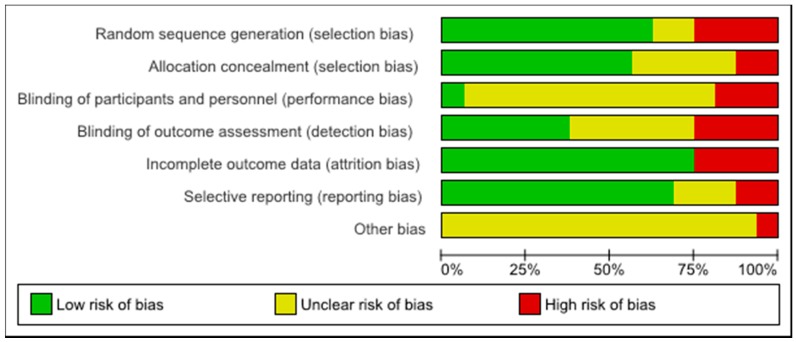
Risk of bias graph: assessment regarding each risk of bias item across all included studies.

**Figure 3:**
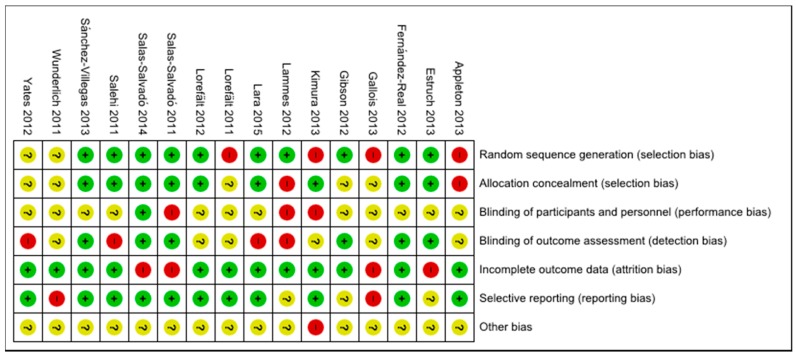
Risk of bias summary: assessment regarding each risk of bias item for each included study.

The quality of the included studies was assessed in terms of risk of bias in this systematic review. Figure 2 shows the risk of bias across all included studies. The low risk of bias (above 50% of the studies) were due to adequate random sequence generation and allocation concealment (selection bias), sufficient outcome data (attrition bias), and adequate reporting of results (reporting bias). Apart from other bias, blinding of participants (performance bias) was the one with the higher unclear risk. Although high risk of bias was identified in each domain across all included studies, it was of relatively small magnitude. Figure 3 shows the risk of bias summary, including assessment of each risk item in each study. None of the studies were assessed as fully low risk of bias, and one study was assessed as unclear or high risk regarding different domains [35]. Nevertheless, in seven studies, more than half of the domains were assessed to be as low risk of bias [20,31,32,36,38,41,44].

### 3.4. Effect of Interventions

Table 2 shows the main measurable outcome of each included study by intervention type.

#### 3.4.1. Dietary Educational Interventions

Seven studies investigated the effect of a dietary educational intervention on healthy eating among older people (Table 2) [33,34,35,36,39,43,44]. These interventions consisted of dietary education sessions and of counselling, focusing on the benefits of a healthy diet. Three studies [33,36,43] reported significant differences between the intervention and control groups in terms of fruits and vegetables (FV) frequency and actual intake, nutrition status or stages of change. Variables such as self-efficacy, perceived benefits, and barriers were taken into account in two studies [36,43]. The remaining studies in this category either showed no significant effect by giving individual nutrition advice [34] or only presented positive results within groups when compared with baseline [35,37,44].

The study by Kimura et al. [33] aimed to increase older people’s dietary variety by giving lectures on practising good dietary habits, to lower further their risk of high-level function decline. Compared with the baseline, participants in the intervention group improved their food intake frequency (six food groups), food frequency score, dietary variety score, and self-rated health. A significant difference between the intervention group and control group was shown in the percentage of participants scoring 1–3 in terms of dietary variety score.

The study by Wunderlich et al. [39] investigated the effect of dietary education on older people’s nutrition status by comparing a congregate meal (CGM) group and home delivered meal (HDM) group. Nutrition status improved significantly as a result of meal provision at home (HDM), while other food behaviours improved slightly in both groups (fruit intake and meals consumption) [39]. Yates et al. [43] reported that tailored newsletters together with family support and counteracting perceived barriers improved older people’s healthy eating behaviour compared with the standard group.

Intervention from the study by Salehi et al. [36] consisted of education sessions of increasing FV intake. As a result, people in the intervention group reported higher FV intake, higher perceived benefits of FV intake, higher self-efficacy, and lower perceived barriers.

Two studies did not show significant change of food intake between the intervention and control groups, but differences were identified within each group when compared with the baseline [35,44]. At the first follow-up of the study by Gallois et al. [35], daily FV consumption and weekly fish consumption in the intervention group increased significantly when compared with the baseline, but failed to show significance when compared with the control group. Lara et al. [44] found that two different levels of dietary education had no effect on older people’s food intake and quality. However, when these two groups were merged as one and compared with their baseline, fish intake and Mediterranean diet score improved notably [44].

#### 3.4.2. Meal Service Interventions

Four of the identified studies used the provision of meal services to facilitate older people’s healthy eating (Table 2) [21,32,37,40].

When the concept of repeated exposure to plant-based foods was applied through fruit tasting sessions, a notable increase of fruit consumption was found among those initially classified as ‘low fruit’ group after receiving fruit exposure for five or more times, but not in the control group (receiving only one exposure). Liking did not change over time among repeated exposure groups, but compared with novel fruits, more liking of familiar fruits was found in the participants. A similar effect of fruit exposure on both fruit and vegetable consumption among low-fruit consumers was also identified in this study [37].

Gibson et al. [21] investigated the relationship between FV intake and immune function among older people. The intervention group was required to consume FV ≥ 5 portions/day while the control group was assigned to have normal diets (FV ≤ 2 portions/day). Compared with the 2-portions/day group, older participants who consumed five or more portions per day had a higher antibody binding to pneumococcal capsular polysaccharide [21].

Older people’s nutrition status, body weight, and energy intake improved significantly after receiving the individualized meals from trained staff [32,40]. In addition, Lorefält et al. reported that those in the intervention group, who improved in their nutritional status, used mainly primary health care after one year and did not require specialized care or hospitalization [40].

#### 3.4.3. Multicomponent Interventions

All of the studies in this category had an RCT design and consisted of three allocations. The control group only received low-fat food (following US dietary recommendations); the MD + EVOO group received a Mediterranean diet (MD) enriched with olive oil; and MD + NUTS group received Mediterranean diet enriched with nuts (Table 2) [20,31,38,41,42]. Diet quality was evaluated based on adherence to MD using a validated score. Adherence to the MD increased by around two score points (*p* < 0.001) during the intervention and this increase was associated with 30% lower incidence of type II diabetes mellitus (T2DM) [41]. Combining interventions (MD + EVOO and MD + NUTS) yielded a 52% lower incidence of T2DM [20]. For older participants with high cardiovascular risk, the Mediterranean diet enriched with olive oil was associated with high serum osteocalcin concentrations, indicating a benefit for bone [31]. Furthermore, for participants with T2DM, the intervention (MD + NUTS) additionally decreased the incidence of depression [38]. The improvement of diet quality measurable through the adherence to the MD showed additional cardio-protective benefits [42], resulting in an approximate 30% reduction in cardiovascular risk [42].

## 4. Discussion

This systematic review involved the latest studies and consolidated information on healthy eating interventions targeting older people, filling a previously identified gap in knowledge [22]. In addition, this study focused on daily diet (actual food consumption) instead of dietary supplements. In a previous review [25], the outcome measures of included studies were FV intake, food variety, and older people’s health conditions, which inspired the search for the present review. In this systematic review, and in agreement with existing data [25], dietary education as a common and well-developed method contributed somehow to improve older people’s healthy eating when evaluated in terms of food intake, nutrition status, and eating habits. Four dietary educational studies in this review showed either no significant effect between groups [34] or only positive results within groups when compared with the baseline [35,37,44], which was congruent with previous researches [45,46,47,48,49] (not included in this systematic review because of publication date). Provision of different levels of nutritional information (standard information at control and nutrition education intervention) seems to have the same effect, questioning the need for the additional effort made by educational intervention. Hence, these findings should be treated with caution. Reasons for lack of effect may lie in the compliance procedure or individual differences. For instance, people with poor digestion may have lower energy even though they received high energy food. Thus, randomization, participants, and sample size are critical issues and should be seriously taken into consideration when carrying out a similar type of intervention [25].

Meal service intervention as a direct way has successfully improved older people’s FV intake, as well as their nutrition status and health condition. Nevertheless, serving meals to promote healthy eating was not consistently effective for all older participants in the same intervention group. For instance, repeated fruit exposure to increase fruit and vegetable intake was only found to be effective in low-fruit consumers, but not in general fruit consumers [37].

Similar results were also shown in a previous study [50] that aimed to investigate the effect of home-delivered meal programs on older people’s diet behaviour and nutrition status. In the review of Zhu et al. [50], eight studies were selected for synthesis and only two RCTs were included [51,52]. Two studies did not show any effect of home meal-delivered meals on weight and energy intake reduction or diet and nutrition improvement among older participants [52,53]. The remaining were studies with positive results in nutrition status or nutrition-related behaviour, but half of them were cross-sectional designs, lacking a follow-up study to test the long-term effect.

In this review, all identified studies in the category of multicomponent interventions were under the same project, PREDIMED, with the objective of investigating the effect of a Mediterranean diet on older people’s physical condition. As a widespread and nutritional diet, the Mediterranean diet carries considerable weight in European countries and is closely associated with higher life expectancy [54]. Studies in this field all presented a positive effect on reducing risk of chronic disease by promoting the Mediterranean diet and giving personalized dietary advice to participants, suggesting a direction of designing healthy meals for older people. In addition, the large sample size of these studies strengthened the perceived value of the interventions.

Most dietary interventions have obtained modest success in their results, whether measured as healthy eating index or specific weight loss [55]. Although food choices are not rational, previous interventions were based on the expectation that a rational and informed food choice made by consumers was healthy and sustainable [56]. Consumers’ food choice, however, often falls within the ‘automatic’ category and when it happens within an obesogenic environment, further hinders compliance with recommendations. The effect of small changes towards a healthier diet could be translated into longer life expectancy and better quality of life [57]. For instance, increasing the daily servings of fruits and vegetables, using extra virgin olive oil as salad dressing, snacking with nuts and other dry seeds, drinking water instead of sugar-sweetened beverages or reducing the red meat intake to once per week or less could be an effective way to improve consumers’ diet quality [58,59]. Transferability of the MD to other cultures and circumstances could be successful if it includes a substantial reduction (or even total avoidance) of elements that are fully in opposition to the concept of the traditional MD [59].

However, it should be noted that these multicomponent interventions emphasized the effect of the Mediterranean diet on reducing disease risk instead of creating strategies for developing actual eating habits. On the other hand, compared with the Mediterranean diet, other regional diet interventions have been investigated less and should be considered seriously. For instance, it has been proven that the Nordic diet increased physical performance and may be adopted to prevent type-2 diabetes [60,61]. Therefore, additional studies are needed to investigate the effect of other types of diet to change older people’s eating behaviour. Another example of a healthy diet pattern that can be put into practice for the older consumer is the Dietary Approaches to Stop Hypertension (DASH), consisting of a generous intake of foods of plant origin and limited consumption of lean meat, fish and poultry (2 times/week), fats, and sweets. [62]. A healthier diet for older consumers could be achieved by changing daily diets whether at home or through foodservices.

The major strength of this systematic review was the gathering of the latest research on interventions towards healthy eating in older people’s daily life. Instead of studying the effect of medical supplement treatment or combination with physical activity on older people’s nutrition status and health behaviour, this systematic review focused more on changing older people’s eating behaviour or improving their health condition by promoting a healthy diet. Included studies provided positive evidence that interventions for promoting healthy eating benefited the older people’s eating behaviour in terms of FV consumption and health status in terms of the reduced incidence and risk of chronic disease and improved nutrition status. Moreover, it provides useful information for improving older people’s living standards through simple dietary advice or interventions.

However, this systematic review still had limitations to be acknowledged, even if the majority of included studies performed well and can be applied to promote healthy eating. First of all, time of publication was limited to articles published in the past five years. The major reason for this constraint was that this review’s objective was to collect the latest research and provide evidence of promoting healthy eating addressing older people specifically.

Second, this systematic review failed to run a meta-analysis due to the study quality and high heterogeneity of included studies. High heterogeneity hindered this review to provide more information for further analysis. Moreover, quality assessment of included studies in terms of risk of bias should be considered with caution as it was assessed by the reviewers’ subjective views. Seven studies showed moderate quality [20,31,32,36,38,41,44], but none of the studies was fully judged as low risk of bias in all domains, which may be caused by limited study conditions and less strict intervention operation. In addition, some studies’ data were collected from participants’ self-reports, which weakened the effect of intervention and may lead to high risk of bias [36,43,44].

Inclusion and exclusion criteria in this systematic review were another reason that limited the findings. Although some other types of interventions regarding healthy eating for the older person can be identified, they did not meet the inclusion criteria for this systematic review (study design, length of the intervention, use of conventional foods, and not supplements or fortified foods). For instance, one study found dietary supplementation or fortification of conventional foods significantly improved older people’s energy and protein intake [26], but were not identified in this article. Other previous research investigating the effect of meal time interventions on nutritional outcomes among older people [63,64] were also excluded. Interventions included food improvement by adding sauce to increase energy intake and dining environment changes. However, those studies were not identified in our systematic review because of the limitation of publication year and the target group [63,64].

Above all, the dietary interventions identified in this systematic review were very limited and only classified into three categories. More innovative methods should be explored in the future to update current studies. For instance, nudging as a behaviour-related strategy has been applied to change people’s eating behaviour, with the aim of influencing people’s choice and changing their behaviour based on many options or little interference in economic incentives in a predictable way [65,66,67].

However, in this field, few studies were found targeting older people’s healthy eating. In the future, exploring and upgrading the local dish regarding different regions, and making use of modern information technology and nudging strategies for studying eating behaviour could be acceptable approaches to alter older people’s eating habits.

## 5. Conclusions

This systematic review provided the latest diet interventions on promoting healthy eating among older people across multiple countries, especially in the EU. Effective dietary education, meal service, and multicomponent dietary interventions (e.g., improving adherence to the Mediterranean diet score by providing olive and nuts) increase older people’s FV intake, eating variety, and improved their physical conditions and nutrition status. Diet changes by following the above interventions may promote older people’s healthy eating and improve the quality of life. However, the types of included interventions were very limited, and the quality of included studies was moderate or lower. Further research is needed to enhance the current knowledge by incorporating local diet patterns, modern information technology, and new strategies such as the nudging concept in intervention studies related to older people’s dietary choices.

## Figures and Tables

**Table 1:** Characteristics of the included studies.

Study	Country	Age (Years)	Setting	Sample Size	Study Design	Description of Intervention	Comparison	Duration	Outcome Measures
Fernández-Real 2012 [31]	Spain	55–80 ^1^	PREDIMED study centre	127	RCT	Participants were randomly assigned to the MD + EVOO and MD + NUTS group; dietitians gave personalized dietary advice to participants corresponding to different diets	Control group (low-fat diet)	2 years	Total osteocalcin; procollagen 1 N-terminal propeptide levels; homeostasis model assessment-β-cell function.
Lorefält 2012 [32]	Sweden	83.8 ± 7.7 ^2^	Residential homes	67	Within-subjects design ^3^	A multifaceted intervention model including education on both theoretical and practical issues for staff; individualized snacks were served to the residents	Participants were their own controls	1 year	Energy intake; Body weight; MNA score; length of night-time fasting
Kimura 2013 [33]	Japan	65–90 ^1^	Community centre	Baseline: 141 Intervention: 92	Cluster-RCT	Consisted of a general lecture on the importance of dietary variety and five educational sessions.	The control group was subsequently provided with the same program as a crossover intervention group	3 months	Food intake; frequency score; dietary variety score; self-rated health; appetite; TMIG Index of Competence
Gibson 2012 [21]	UK	65–85 ^1^	Residential area	82	RCT	Intervention group: FV intake ≥5 portions/day	Normal diet (FV intake ≤2 portions/day)	16 weeks	Changes of FV intake (Mean ± SD); antibody assessment
Lammes 2012 [34]	Sweden	≥75 ^1^	Elderly research centre	Baseline: 95 Intervention: 79 follow-up: 64	RCT (Pilot study)	Three types of intervention (1) Nutritional intervention: individual dietary counselling and estimation of each participant’s energy needs (2) Physical training (3) Combined nutritional and physical intervention	General advice regarding diet and physical training	1 year	Energy intake; resting metabolic rate; fat-free mass
Gallois 2013 [35]	Germany	≥57 ^1^	Low socio-economic status district: community partners’ institution, churches and mosques	Baseline: 423 Intervention: 369	Quasi- Experimental Study	The intervention comprised seven sessions. In each session, older participants discussed health topics and received counselling aid; standard health information on physical activity and nutrition, and cooking recipes were handed out at the end of each session	The control group only received standard health information and cooking recipes by post	1 year	Changes of FV intake; dairy product and fish intake (Mean ± SD)
Salehi 2011 [36]	Iran	64.06 ± 4.48 ^2^	Elderly centre	Intervention group: 200 Control group: 200	Quasi-Experimental Study	Participants received four weekly sessions including introduction, stages of change for FV intake, reinforcement of second session, and barriers anticipated and overcome	Control group: general health education	4 weeks	Changes in food intake (mean serving/day); stage transitions; self-efficacy; perceived benefits and barriers
Appleton 2013 [37]	UK	≥65 ^1^	Community-based church and social group	95	Quasi-RCT	Participants were randomized to receive five (*n* = 38) or five plus (*n* = 18) exposures of fruit over a 5-week period	One-time exposure	5 weeks	Fruit intake and liking; FV intake and liking (Mean ± SD)
Sánchez-Villegas 2013 [38]	Spain	Men: 55–80 women: 60–80 ^1^	Primary care centre	MD + EVOO: 1446 MD + NUTS: 1293 Control group: 1184	RCT	Participants were randomly assigned to the MD + EVOO and MD + NUTS group, and received intensive education on MD	Low-fat diet including recommendations to reduce all types of fat intake	3 years	Risk of incidence of depression
Wunderlich 2011 [39]	USA	≥60 ^1^	Congregate and home delivered meal locations	Baseline: 476 Intervention: 355	Quasi-Experimental Study	CGM (congregate meal) participants: regular topical nutrition education and counselling in a classroom format with cooking demo, discussion, and handouts	The HDM (home delivered meal): participants only received the printed material (same handouts) and counselling by telephone	2 years	FV intake (%); Nutrition risk score; Meal intake/day
Lorefält 2011 [40]	Sweden	83–86 ^1^	Residential homes	Intervention group: 42 Control group: 67	Quasi-Experimental Study	A multifaceted intervention design was adopted; nutritional status of older participants was measured by MNA; individualized meals were provided to the residents based on the results of the MNA	Only received education on how to measure MNA; residents from the control group followed the usual meal routines	3 months	Body weight; MNA score; cost of health care
Salas-Salvadó 2014 [41]	Spain	Men: 55–80 women: 60–80 ^1^	Primary care centre	MD + EVOO: Intervention: 2543 (follow up: 1154) MD + NUTS: Intervention: 2454 (follow up: 1240) Control group: 2450 (follow up: 1147)	RCT	Participants were randomly assigned to the MD + EVOO and MD + NUTS group; dietitians conducted individual and group dietary training sessions to provide information on typical Mediterranean foods, seasonal shopping lists, meal plans, and recipes	Received only a leaflet describing low-fat diet	7 years	Incidence of diabetes; MD adherence; MD score ^4^
Estruch 2013 [42]	Spain	Men: 55–80 women: 60–80 ^1^	PREDIMED study centre	MD + EVOO: 2543 MD + NUTS: 2454 Control group: 2450	RCT	Participants were randomly assigned to the MD + EVOO and MD + NUTS group; dietitians ran individual and group dietary-training sessions at the baseline visit and quarterly thereafter	Control group received small non-food gifts	4.8 years	Rate of cardiovascular events
Salas-Salvadó 2011 [20]	Spain	Men: 55–80 women: 60–80 ^1^	PREDIMED study centre	418	RCT	Participants were randomly assigned to the MD + EVOO and MD + NUTS group; dietitians gave personalized dietary advice to participants	Received only a leaflet describing the low-fat diet	5 years	Incidence of diabetes
Yates 2012 [43]	USA	Women: 50–69 ^1^	Rural research offices	225	Cluster-RCT	A repeated-measures experimental design: intervention group received tailored newsletter	Group received standard newsletter	2 years	Self-efficacy; benefits of healthy eating; family support; perceived barriers
Lara 2015 [44]	UK	≥50 ^1^	Human nutrition research centre	23	RCT	Evaluated the feasibility of a three-week brief MD intervention with two levels of dietary advice; Level 2: EGS and received additional support	Level 1: only attended an EGS	3 weeks	Food intake (Mean ± SD); MD score; cost of adopting an MD/day

^1^ Age range; ^2^ mean age; ^3^ Within-subjects design: each participant was exposed to all the different treatments, including the control. ^4^ MD score: to estimate participants’ adherence to the Mediterranean diet, and high score is positively associated with high intake of Mediterranean diet. Abbreviations: FV = Fruits and vegetables, MD = Mediterranean diet, MD + EVOO = Mediterranean diet enriched with extra virgin olive oil, MD + NUTS = Mediterranean diet enriched with nuts, EGS = Educational group session, MNA = Mini nutritional assessment, BMI = Body mass index, TMIG = Tokyo Metropolitan Institute of Gerontology, CGM = Congregate meal, HDM = Home delivered meal.

**Table 2:** Effect of dietary interventions on older people by intervention type.

Study	Main Outcomes
**Dietary educational interventions**	
Kimura 2013 [33]	Percentage of participants who scored 1–3 regarding the dietary variety showed a significant difference (*p* = 0.041) between the intervention group and control group. Improvement rate of self-rated health did not show a significant difference between the control group and intervention group. Compared with the baseline, there was a significant increase of post-intervention food intake frequency in the intervention group in the following items: daily consumption of meat +19.3%, *p* = 0.002; fish/shellfish +8.7%, *p* = 0.02; eggs +8.8%, *p* = 0.01; potatoes + 10.5%, *p* = 0.019; fruits +10.5%, *p* = 0.029; seaweed +22.8%, *p* = 0.001; an increase in food frequency score (mean +2.4 points, *p* < 0.001); in dietary variety score (mean +1.2 units, *p* = 0.001); in self-rated health (7% were in ‘not good’ category, *p* = 0.003). In the control group, there was no significant difference between the baseline and post-intervention. Appetite and TMIG Index of Competence score did not change between baseline and post-intervention in both groups.
Lammes 2012 [34]	Individual nutrition counselling had no effect on energy intake, resting metabolic rate, and fat-free mass.
Gallois 2013 [35]	No significant differences were found between the control group and intervention group at the first follow-up. Compared with the baseline, except for dairy product consumption, there were significant increases of daily fruit and vegetable consumption (+23 participants reached the recommended level, *p* = 0.04) and weekly fish consumption (+33 participants reached the recommended level, *p* = 0.04) in the intervention group at the first follow-up. Similar results were shown in the control group. Dairy product consumption did not present any changes.
Salehi 2011 [36]	Compared with the control group, the intervention group showed significant increase of FV intake (mean +1.3 servings/day, *p* = 0.001), perceived benefits (mean +9.44 points, *p* < 0.001) and self-efficacy (mean +5.64 points, *p* < 0.001), but lower perceived barriers (mean −6.9% points, *p* < 0.001) at post-test assessment. Compared with the control group, a larger percentage of older people in the intervention group moved from precontemplation to contemplation/preparation and action/maintenance stages (*p* < 0.0001), and from contemplation/preparation to action/maintenance stages (*p* = 0.004).
Wunderlich 2011 [39]	Nutrition education and counselling improved nutrition risk scores significantly in HDM group (mean −2 points, *p* < 0.01) but not in CGM group (mean −0.44 points, *p* = 0.14). Slight improvements in nutrition behaviours were found in HDM group eating ≥2 meals (+5.6%) and CGM group eating ≥5 servings of fruits and vegetables (+3.4%).
Yates 2012 [43]	Self-efficacy and benefits of healthy eating did not change significantly over time between groups (tailored newsletter group and standard group). At the end of intervention, the tailored newsletter group got significantly more family support (b = −0.289, β = −0.366, z = 2.4, *p* < 0.05) and a less perceived barrier than the standard group (b = 0.14, β = 0.369, z = 2.42, *p* < 0.05).
Lara 2015 [44]	No significant differences were shown in group 1 (educational group session on MD) and group 2 (educational group session on MD with additional support). Compared with the baseline, mean fish intake (+25.9, *p* = 0.01) and mean MD score (+0.6 points, *p* = 0.05) increased significantly when analysing the combined group, but no significant difference was found in food intake cost.
**Meal service interventions**	
Lorefält 2012 [32]	MNA score significantly increased after 3 months’ intervention (mean +1.3 points, *p* = 0.01) and it was maintained after 9 months; weight (mean +1.9 kg, *p* = 0.0001) and energy intake (me-an +376 kcal, *p* = 0.0001) increased significantly during the whole period; length of night-time fasting decreased (after 3 months’ intervention: −0.8 h, *p* = 0.0001, after 9 months’ intervention: −0.6 h, *p* = 0.01), but not to the recommended level.
Gibson 2012 [21]	After 16 weeks, the change in FV intake showed a significant difference (*p* < 0.001) between 2-portion/day group (0.4 portions/day) and 5-portion/day group (4.6 portions/day); antibody binding to pneumococcal capsular polysaccharide increased more in the 5-portion/day group than in the 2-portion/day group (geometric mean +1.4, *p* = 0.005).
Appleton 2013 [37]	At week 1, except for liking familiar fruits, no differences were found in other measures between any groups. In low fruit intake consumers, a significant increase of fruit intake was found in the repeated groups (five or five plus exposures to fruit: mean 0.6 and 0.8 portions/day, respectively, *p* < 0.01), but not in the one-time fruit exposure group (mean 0.3 portions/day, *p* = 0.78) at week 1.Similar results were found over the whole experiment duration. No differences were found between the five and five plus exposure groups (*p* = 0.31). No changes in liking were identified over time or between repeated exposure groups, but familiar fruits showed an increase in liking (*p* < 0.01) than novel fruit products and dishes. Similar exposure effects were also shown on FV intake and liking (FV intake increase in the repeated exposure group: *p* = 0.01, in one-time fruit exposure group: *p* = 0.97).
Lorefält 2011 [40]	After 3 months, MNA score (malnourished −14.3%, *p* < 0.01) and body weight (mean +2.7 kg, *p* < 0.001) increased significantly in the intervention group compared with the control group; cost of primary health care occupied about 80% of the total median cost in the intervention group and about 55% in the control group.
Multicomponent Interventions	
Fernández-Real 2012 [31]	The total osteocalcin (mean +1.5 ng/mL, *p* = 0.007), procollagen 1 N-terminal propeptide levels (mean +71.6 ng/mL, *p* = 0.01) and homeostasis model assessment-β-cell function (mean +11.5 units, *p* = 0.01) increased significantly in MD +EVOO group, but not in the MD +NUTS group (*p* = 0.32) and control groups (*p* = 0.74) after the intervention period.
Sánchez-Villegas 2013 [38]	Risk of depression in participants assigned to MD + NUTS was inversely associated with the control group, but not significant. When analysis targeted participants with type 2 diabetes, risk of depression showed significant reduction in participants assigned to MD + NUTS compared with the control group (−41%, *p* = 0.04).
Salas-Salvadó 2014 [41]	During follow-up, mean scores of adherence to the Mediterranean diet increased in the Mediterranean diet group compared with the control group (mean +around 1.5–2 points, *p* < 0.01); proportion of participants with a Mediterranean diet score of 10 or higher was larger in the Mediterranean diet group than in the control group (*p* < 0.010) over the whole duration. Rates of diabetes cases in MD + EVOO group, MD + NUTS group and control group were 16.0, 18.7, and 23.6 cases per 1000 person years, respectively. Multivariate-adjusted hazard ratios for MD + EVOO group and MD + NUTS group were 0.60 and 0.82 compared with the control group. When considering the two MD groups together, diabetes incidence was reduced (−30%) compared with the control group.
Estruch 2013 [42]	Compared with the control group, multivariate-adjusted hazard ratios in MD + EVOO group and MD + NUTS group were 0.70 and 0.72, respectively. Cardiovascular risk was reduced (around 30%) by MD + EVOO or MD + NUTS.
Salas-Salvadó 2011 [20]	Diabetes incidence in MD + EVOO group, MD + NUTS group, and control group were 10.1%, 11.0%, and 17.9%, respectively. When considering the two MD groups together, diabetes incidence reduced (−52%) when compared with the control group.

Abbreviations: FV = Fruits and vegetables, MD = Mediterranean diet, MD + EVOO = Mediterranean diet enriched with extra virgin olive oil, MD + NUTS = Mediterranean diet enriched with nuts, EGS = Educational group session, MNA = Mini nutritional assessment, TMIG = Tokyo Metropolitan Institute of Gerontology, CGM = Congregate meal, HDM = Home delivered meal.
